# Chinese football violence: An extended theory of planned behavior model to predict fans’ violent behavioral intentions

**DOI:** 10.3389/fpsyg.2022.977497

**Published:** 2022-10-31

**Authors:** Yuge Tian, Chao Ma, Zhenguo Shi

**Affiliations:** School of Physical Education, Shandong University, Jinan, China

**Keywords:** football fans, violent behavioral intentions, theory of planned behavior, subjective norms, deindividuation, attitude, perceived behavioral control, China

## Abstract

This study introduced deindividuation (DI) variables and constructed a mechanism model of football fans’ violent behavioral intentions (FVBI) in China based on theory of planned behavior (TPB). Specifically, taking Chinese football fans as the research subjects, we used a structural equation model (SEM) to explore the specific effects of subjective norms (SNs), perceived behavioral control, DI, and attitude (AT) on violent behavior intentions. Our results showed that SNs (β = -0.132, *P* < 0.01) had a significant negative predictive effect on FVBI, while perceived behavioral control (β = 0.297, *P* < 0.01), DI (β = 0.239, *P* < 0.01), and AT (β = 0.416, *P* < 0.01) had a significant positive predictive effect. However, we found that AT was the most important factor that predicted the FVBI and played a mediating role between SNs and FVBI, between perceived behavior control (PBC) and FVBI, and between DI and FVBI as well.

## Introduction

Football (also known as soccer) is the most popular spectator sport in the world, and has begun to attract even more fans to attend games in person at stadiums in recent years. In China specifically, the average audience for a football match of Chinese Football Association Super League reached 15,000, and the number of football fans in China now exceeds 100 million (National Development and Reform Commission, 2016). The number of penalties handed out by the Chinese Football Association for incidents of fan unrest between 2015 and 2019 was three times higher than those handed out between 2010 and 2014 (Chinese Football Association, 2016). Although the phenomenon of Chinese football fan violence has a short history compared to the long-established British football hooligans (Newson, 2019), and does not carry anti-social attributes compared to the Brazilian football hooligans who are known for their anti-social and serious violence (Lopes, 2013), with the continuous development of Chinese football, Chinese football fan violence is also gradually developing in the direction of scale and organization, which seriously affects national and social stability (Liang, 2018). Hence the prevention and containment of violent football fans is particularly important. One way to help with this may be to try to predict when groups of fans are about to become violent. Behavioral intention was one of the most important predictors of actual behaviors (Ajzen, 2002) and could therefore be useful in exploring the influence mechanism of Chinese football fans’ violent behavior intentions (FVBI).

Modern football fans’ violence originated in the United Kingdom (Leeson et al., 2012) and then gradually spread across Europe, taking the form of English hooligans and ultras in Southern and Eastern Europe. The hooligan forms were less concerned with the visual, less integrated with the football clubs, and less politically minded than the Southern and Eastern European ultras. Notable was France, which had English hooligans in the north and European ultras in the south (Mignon, 2002). Fans’ violence has led to numerous casualties, such as the 1985 Hessel tragedy (Pyta, 2015), the Euro 2016 clashes between British and Russian fans (CCTV, 2016), and the England fan riots at the 2021 European Championship final (CCTV, 2021), which have also caused incalculable economic and reputational damage to the region.

Several scholars have already discussed the internal mechanism of violent behavior based on different research perspectives. Simons and Taylor (1992) put stadium spectator violence in a social environment and proposed a social psychological model of stadium spectator violence, including socio-economic conditions, and social norms; key factors such as identification, deindividuation (DI), group consciousness, dehumanization of the opponent, leadership; gameplay factors such as score changes and competition; and other factors such as alcohol and audience density. Knapton et al. (2018) have assessed the effects of a “need to belong” and rejection sensitivity on football fans’ willingness to engage in violence from a social personality perspective. In addition, the analysis of the causes of fan violence based on social practice theory has opened up a new perspective for research in this area (Xing et al., 2019). Although the underlying mechanisms of fan violence may vary in related studies due to the various cohorts of fans that were studied, changes in the environment inside and outside the stadium, and differences in research perspectives, all of these previous studies have emphasized that the generation of fan violence is a complex process and that its underlying mechanisms are influenced by a combination of factors. Moreover, the theoretical discussion on the core elements of internal mechanism of fan violence from the perspectives of sociology and psychology also lays a certain foundation for the subsequent quantitative research. For example, Liang (2018) constructed a dual path model of cognition and emotion for the violent behavior of football fans on the basis of cluster behavior theory and carried out empirical tests after conducting a survey, and Shi and Shi (2015) used dynamic methods and numerical simulation to depict the dynamic evolution law of spectator violence in stadiums.

Elsewhere, in order to investigate the violent behavior of fans, priority is given to understand their intentions (Elliott et al., 2007). In the field of behavioral intention research, the theory of planned behavior (TPB) is considered to be one of the most powerful explanatory models (Armitage and Conner, 2001), and many empirical studies have also shown that the TPB can explain various behavioral intentions (George, 2004; Norman and Hoyle, 2004). However, empirical studies on fans’ intentions for violent behavior from the perspective of TPB are relatively scarce. What factors influence football fans’ violent behavior intentions and then trigger violent behavior? What are the mechanisms of action between these factors?

Existing researches does not answer these questions. In view of this, the present analysis framework was based on TPB and external variables were introduced to expand our initial TPB model. Then, a Structural Equation Model (SEM) was built to explore the mechanism of football fans’ violent behavior intentions, which may lead to effective ways to prevent such violence in the future.

### The theory of planned behavior

As an important theoretical model for the study of individual behavior, the TPB model has been successfully applied in various fields as psychology (Morrison et al., 2010; Chan et al., 2020), sociology (Mannetti et al., 2002; Qiu, 2017), management (Ramsey et al., 2008; Teng et al., 2015), sport sciences (Nigg et al., 2009), and has been shown to improve the explanatory and predictive power of research on behavior significantly. Seeing that, the violent behavior of football fans belongs to the category of typical and representative individual behavior decisions (Liang, 2017), the TPB was applied as the basic analysis framework of this study. The TPB was proposed by Ajzen (1991) to explain the general decision-making process of individual behavior based on the theory of expected value (WenTing and GuangRong, 2008), and this theoretical model involves four variables, attitude (AT), subject norms (SN), perceived behavior control (PBC), and behavioral intention, where the first three variables can also have an impact on behavioral intention (Ajzen, 1991). Combining the work of existing studies, football fan violence was defined as abusing, insulting, provoking, or beating by spectators of football matches with mild violence (verbal, body language, and slogans) or intense violence (physical contact and confrontation.) against fans, players, coaches, or other staff members of other teams during the pre-match entrance, during the match or post-match departure (Young, 2013; Spaaij, 2014; Liang, 2018). Football fans’ violent behavioral intentions is the degree to which football fans are willing to put in efforts to commit violent acts (Liang, 2018). Subjective norms (SNs) were defined as the social pressure from their relatives and friends that football fans perceive as motivating them to develop positive behavioral norms when making decisions on whether to commit violent acts and AT as football fans’ comprehensive evaluation of the integrity of committing violence (Ajzen, 1991). PBC was defined as the perceived ease with which football fans commit violent acts (Ajzen, 2002).

Spaaij (2014) indicated that football fans’ intention to act violently was influenced by the social relationships around them. Young (2015) believed that football fans’ ability to control violent behavior could regulate their intention to act violently. At the individual psychological level of football fans, AT was an important psychological factor of football fans’ intention to act violently (Braun and Vliegenthart, 2008). Additionally, according to the TPB, AT, SNs, and PBC were all effective predictive variables for behavioral intentions (Ajzen, 1991). Based on this, the following hypotheses were proposed.

**H1:** Attitude has a positive predictive effect on football fans’ violent behavioral intentions.

**H2:** Subjective norms have a negative predictive effect on football fans’ violent behavioral intentions.

**H3:** Perceived behavioral control has a positive predictive effect on football fans’ violent behavioral intentions.

Subject norms are not only effective predictors of driving behavioral intention but also have a significant impact on AT (Al-Rafee and Cronan, 2006; Xiao, 2019), and this view is strongly supported by persuasion theory and cognitive dissonance theory in the field of social psychology. According to persuasion theory, persuasive suggestions of others in a group can influence the formation of individual ATs to a certain extent (Eagly and Chaiken, 1993), and cognitive dissonance theory points out that in order to cater to group norms, individuals in a group may consciously change their ATs (Festinger, 1962). In addition, given that AT has a significant impact on behavioral intention, some studies further pointed out that AT mediated the relationship between SNs and behavioral intention (Bai and Lin, 2021). Accordingly, the following additional hypotheses were proposed.

**H4:** Subjective norms have a negative predictive effect on attitude.

**H4a:** Attitude plays a mediating role between subjective norms and football fans’ violent behavioral intentions.

Some scholars have pointed out that PBC has an impact on AT (Qiu, 2017; Wang et al., 2018). The research results of Deng (2012) on consumers’ ethical purchasing intentions showed that the lower the control degree of consumers’ perceived behavior on ethical shopping, the lower their ethical purchasing AT would be. Qiu (2017) also showed through empirical research that AT had at least a partial intermediary effect between PBC and the pro-environmental behavioral intention of outbound tourists. Based on these, the following two additional hypotheses were proposed.

**H5:** Perceived behavioral control has a positive predictive effect on attitude.

**H5a:** Attitude plays a mediating role between perceived behavioral control and football fans’ violent behavioral intentions.

### An extended theory of planned behavior model based on deindividuation

An important assumption of the TPB is that the occurrence of behavior is a rational decision-making process from belief, to belief evaluation, to the generation of behavioral intention, and to the final initiation of behavior (WenTing and GuangRong, 2008). However, the occurrence of behavioral intention is not always based on individual rational decisions and is often the product of multiple factors. Therefore, the TPB model is not perfect, and other specific factors need to be introduced to supplement and improve the research on individual decision making in specific situations (Ajzen, 1991). Football fans’ violent behavior is regarded as a typical cluster behavior (Liang, 2018), and behavioral intention in the stadium atmosphere decisions can be influenced by swarm behavior. Hence, the loss of self-control and being “lost in the crowd” can produce the phenomenon of DI (Zhang, 2008), and ultimately induce violent behavioral intention. The decision-making process of football fans is not only based on the judgment of individual rationality but also affected by the irrational tendency of individuals to deinidividuate in a group situation (Shi, 2004). Therefore, DI is another potential predictive variable for FVBI. Based on this, this study incorporates DI into the TPB model.

Deindividuation is an important theory in social psychology studies on human aggression and refers to the phenomenon of individuals losing their self-perception and significantly self-control in group settings (Festinger, 1952). From the perspective of individuals, the process is one that shifts from personal identity to social identity, with the redefinition of “I” to “we” (Diener et al., 1980), that is accompanied by emotional and impulsive performance. Additionally, under the influence of DI, individuals may react irrationally compared to their individual intentions, and this can manifest in brutality, violence, and the release of language inhibitions (Diener et al., 1976). Based on this, in the context of the football pitch, for the purposes of the present study we defined DI (Zhang, 2008) as the degree to which football fans perceive the loss of their own individuality and the decline of their own behavior control, resulting in abnormal and anti-rule behaviors when making decisions on whether to commit football fan violence.

Many scholars have noticed the effect of DI on football fan violence. For example, Shi (2004) believed that the feeling of DI can greatly increase the possibility of fans’ aggressive behavior in a stadium, and Wang et al. (2008) believed that from the perspective of individual psychology, DI was an important factor for the occurrence of spectator violence. The research of Zhang (2008) also showed that after the three main stages of DI, namely, individual excitement, collective excitement, and social contagion, individual behavior of football fans and group behavior stimulate and reinforce each other, and group behavior can promote and encourage the individual emotion and behavior of football fans until a fanatical state appears. Wang and Wei (2014) also found through empirical research that DI had a significant positive impact on the supportive AT toward group events. Specifically, compared with other variables, in the regression analysis of support AT toward group events, DI had the best predictive effect on support AT toward group events, with an explanation rate (*R*^2^) of 18% (Wang and Wei, 2014). However, according to Identity Fusion Theory, fans also undergo identity fusion in the stadium environment. In identity fusion, others in the group are perceived as unique individuals and individuals do not deindividuate (Swann et al., 2012). A study of British football fans by Newson et al. (2022) showed that highly integrated fans prioritize ingroup altruism over outgroup hostility and were most likely to report altruistic behavior toward hostile fan groups. Only under high threat conditions did older fused fans expect future outgroup hostility. A study of Brazilian football fans also showed that the effect of fusion on violence was not statistically significant for the average fan (Newson et al., 2018). Therefore, whether Chinese football fans deindividuate in the stadium environment and whether DI predicts Chinese football fans’ violent intentions need to be further verified through empirical studies. Based on this, the following three additional hypotheses were proposed.

**H6:** Deindividuation has a positive predictive effect on football fans’ violent behavioral intentions.

**H7:** Deindividuation has a positive predictive effect on attitude.

**H7a:** Attitude plays a mediating role between deindividuation and football fans’ violent behavioral intentions.

**FIGURE 1:**
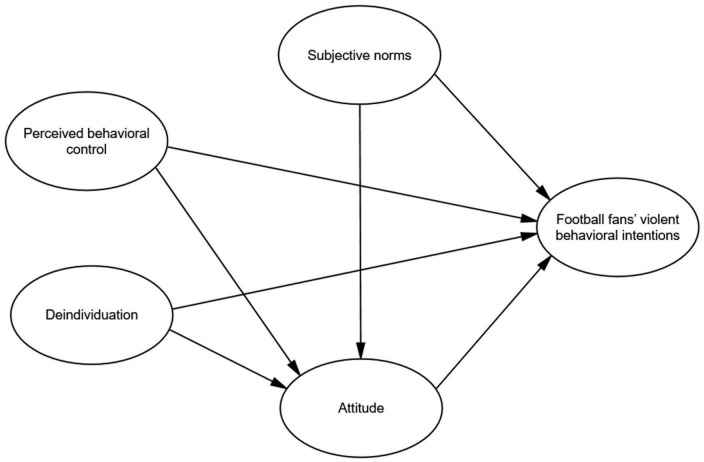
The hypothesis model.

To summarize, the present study constructed a mechanism model of FVBI based on an extended TPB model by introducing of DI variable. The “hypothesis model” is shown as follows (Figure 1). Based on this, it aimed to (1) analyze the relationship between FVBI and SNs, AT, PBC, DI and (2) explore the mediating role of AT in the relationship between SNs and FVBI, PBC and FVBI, and DI and FVBI.

## Materials and methods

### Participants and procedures

Participants completed an anonymous online and offline questionnaire survey. It was addressed only for Chinese football fans. Survey started on October 1, 2021, and was collected until November 21, 2021. Online questionnaire was distributed through the network system of “Questionnaire Star,” which was then promoted by WeChat, QQ, and TikTok. Questionnaire item was set to ask whether the questionnaire filler had been to a live football match to confirm whether the questionnaire filler was a football fan. Offline questionnaires were widely distributed to football fans by research group members near Jinan Olympic Sports Center and Shandong Sports Center, both in Shandong Province, China. Through the convenience sampling method, we selected 441 football fans from the “Football Fans Association” in Beijing, Shanghai, Tianjin, Gansu, and Shandong to participate in the study. Among them, 161 offline questionnaires and 280 online questionnaires were collected. All participants who participated in this study were given a material reward of 5 RMB after completing the questionnaire. In total, 382 valid questionnaires remained after excluding invalid questionnaires, for an effective recovery percentage of 86.7%. Socio-demographic information of participants was shown in Table 1.

**TABLE 1:** Profile of respondents.

Variables	*N*	%
**Gender**		
Male	300	78.5%
Female	82	21.5%
**Age**		
Under 20 years	76	19.9%
21–25 years	202	52.9%
26–30 years	46	12.0%
31–35 years	34	8.9%
36–40 years	9	2.4%
Above 40 years	15	3.9%
**Occupation**		
Students	108	28.3%
Civil servants	74	19.4%
Corporate employees	87	22.8%
Freelancers	51	13.3%
Others	62	16.2%

### Questionnaire design

The questionnaire used in this study consists of three parts. The first part is the preface, which explains the purpose, content, and privacy guarantee of the questionnaire. The second part covers demographic variables, including gender, age, occupation, and education level, and the third part is the subject scale. In addition, we also relied on results from expert interviews on how to design the measurement items of variables related to FVBI. After sorting out and designing the questionnaire items, we distributed 100 questionnaires for pre-survey on the basis of ensuring that each respondent was a football fan, and from this, we collected 96 valid questionnaires. According to the results, we revised unclear and difficult-to-understand items in the original questionnaire and deleted some items using item and exploratory factor analysis. This resulted in the formation of an overall scale consisting of five parts, SNs, PBC, DI, AT, and FVBI (Table 2).

**TABLE 2:** Constructs and measurement items.

Constructs	Items
SN	My relatives and friends around me strongly oppose my violent football fan behavior (SN1).
	My relatives and friends all think I should watch the match civilly (SN2).
	My relatives and friends all think that my violent football fan behavior will have a bad influence on me (SN3).
	If I were to commit football fan violence, I would feel that people around me would have a bad opinion of me (SN4).
PBC	Committing football fan violence is not very difficult (PBC1).
	I am competent enough to commit acts of football fan violence (PBC2).
	I believe that even if I commit certain acts of football fan violence, I will not face serious repercussions (PBC3).
DI	The degree to which I lost my sense of self in the stadium environment (DI1).
	Compared with non-football stadium environments, I am more likely to make excited remarks and take drastic actions to vent my emotions in the stadium environment (DI2).
	My self-evaluation of myself in the stadium environment deviates from my evaluation in the rest of my life (DI3).
	When I participate in cluster behaviors with a group, I am more likely to indulge my words and deeds due to the decline of my individual consciousness (DI4).
AT	Committing football fan violence can set me free (AT1).
	Committing football fan violence can make me relax (AT2).
	It is necessary to commit violence as football fans (AT3).
FVBI	In the future, I may violate the regulations on civilized viewing of matches (FVBI1).
	In the future, I may commit football fan violence in the form of verbal language, body language, and slogans (FVBI2).
	I may commit football fan violence in the form of physical contact or confrontation in the future (FVBI3).

SN, subjective norm; PBC, perceived behavioral control; DI, deindividuation; AT, attitude; FVBI, football fans’ violent behavioral intention.

### Variables measurement

In this study, a more mature scale was used to ensure the questionnaire’s reliability and validity, and the scale was modified to account for the survey’s context. The specific scale items, as well as relevant references, are listed in Tables 2, 3. All variables were evaluated using a 5-point Likert scale, with 1 indicating “strongly disagree” and 5 indicating “strongly agree.” In addition, it is important to explain that fans in China are often urged to watch games in a civilized manner. The “regulations on civilized viewing of matches” in the measurement item FVBI1 refers specifically to the regulations on stadium violence. This is to cover possible forms of violence or new forms of violence that we have not listed other than measurement item FVBI2 and measurement item FVBI3.

**TABLE 3:** Reliability and validity testing of our scale.

Constructs	Items	Factor loadings	Cronbach’s α	CR	AVE	References
SN	SN1	0.708	0.844	0.851	0.590	Han et al., 2010; Song et al., 2014
	SN2	0.741				
	SN3	0.874				
	SN4	0.738				
PBC	PBC1	0.741	0.807	0.813	0.594	Dimmock and Grove, 2005
	PBC2	0.851				
	PBC3	0.713				
DI	DI1	0.581	0.846	0.851	0.593	Festinger, 1952; Tajfel and Turner, 1986; Xu et al., 2016
	DI2	0.821				
	DI3	0.819				
	DI4	0.830				
AT	AT1	0.896	0.898	0.899	0.749	Ragheb and Beard, 1982
	AT2	0.891				
	AT3	0.807				
FVBI	FVBI1	0.874	0.914	0.914	0.779	Liang, 2017
	FVBI2	0.900				
	FVBI3	0.874				

### Statistical analysis

The Harman single-factor model was used to test the common method bias after data acquisition (Zhou and Long, 2004). SPSS 26.0 and AMOS 24.0 were used for data analysis. First, descriptive statistics and reliability and validity tests were performed for all variables in SPSS 26.0. Secondly, a structural model was established in AMOS 22.0 based on research assumptions, and the fitting degree of the model was analyzed and the path analysis was carried out. Finally, Bootstrap method was used to estimate 95% confidence intervals (CI) by repeated sampling 2,000 times to further test the significance of the mediating role of AT in the whole model. If the 95% CI does not contain 0, the direct or indirect effects are considered significant (Hayes, 2009).

## Results

### Common method bias test

Unrotated principal component factor analysis results showed that the variance explanation rate of the first factor was 38.855, less than 50%.

### Reliability and validity testing for the scale

The analysis results for this testing are shown in Table 3. Cronbach’s α coefficient values ranged from 0.807 to 0.914 (all greater than 0.7) and the Composite Reliability (CR) values ranged from 0.813 to 0.914 (all greater than 0.7). The Average Variance Extracted (AVE) values of the latent variables ranged from 0.590 to 0.779 (all greater than 0.5). In addition, in terms of discriminant validity, the square root AVE value of each latent variable was greater than the correlation coefficient between the variable and other variables (Table 4), which passed the discriminant validity test.

**TABLE 4:** The discriminatory validity test of variables.

Constructs	SN	PBC	DI	AT	FVBI
SN	**(0.768)**				
PBC	0.064	**(0.771)**			
DI	0.054	0.570	**(0.770)**		
AT	–0.067	0.648	0.466	**(0.866)**	
FVBI	–0.128	0.694	0.595	0.728	**(0.883)**

The square root of the average variance extracted (AVE) for each construct is denoted in bold and parentheses, while the inter-construct correlations are shown off-diagonally.

### Structural equation modeling analysis

#### Model fit analysis

The fitting degree of the model was tested and the results are shown below: CMIN/DF = 2.575; CFI = 0.956; GFI = 0.919; AGFI = 0.887; IFI = 0.957; TLI = 0.946; RMSEA = 0.064; and SRMR = 0.054. The fitting degree indexes and guidelines of the final model are shown in Table 5.

**TABLE 5:** The structural model fit indices.

Indices	CMIN/DF	CFI	GFI	AGFI	IFI	TLI	RMSEA	SRMR
Guideline	1–3	>0.90	>0.90	>0.80	>0.90	>0.90	<0.08	<0.08
Value	2.575	0.956	0.919	0.887	0.957	0.946	0.064	0.054
Conclusion	Qualified	Qualified	Qualified	Qualified	Qualified	Qualified	Qualified	Qualified

#### The path coefficients test of the hypothesis model

**FIGURE 2:**
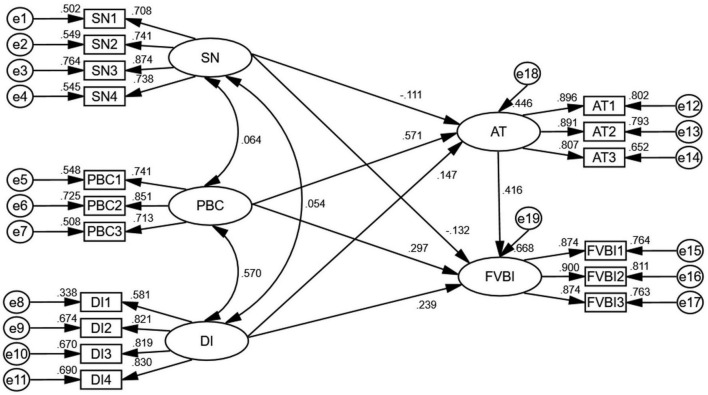
The final path model.

We carried out a path test on our model, and the test results are shown in Table 6. The results showed that H1, H2, H3, H4, H5, H6, and H7 all passed the hypothesis test. Here we saw that the standardized path coefficients for AT, PBC, DI, SNs on FVBI were 0.416, 0.297, 0.239, and –0.132, respectively, and were statistically significant at *P* < 0.05, indicating that AT, PBC, and DI had a positive predictive effect on FVBI. SNs have a negative predictive effect on FVBI. However, the standardized path coefficients for PBC, DI and SNs on AT were 0.571, 0.147, and –0.111, respectively, and were statistically significant at *P* < 0.05, indicating that PBC, DI, and SNs had positive effects on AT. The final calculation results of the SEM are shown in Figure 2.

**TABLE 6:** The path coefficients test of the hypothesis model.

Hypothesis	Path correlation	*B*	S.E.	*T*	*P*	β	Results
H1	Attitude→football fans’ violent behavioral intentions	0.39	0.053	7.366	***	0.416	Supported
H2	Subjective norm→football fans’ violent behavioral intentions	–0.174	0.051	–3.379	***	–0.132	Supported
H3	Perceived behavioral control→football fans’ violent behavioral intentions	0.358	0.078	4.569	***	0.297	Supported
H4	Subjective norm→attitude	–0.155	0.066	–2.344	0.019	–0.111	Supported
H5	Perceived behavioral control→attitude	0.734	0.091	8.076	***	0.571	Supported
H6	Deindividuation→football fans’ violent behavioral intentions	0.239	0.051	4.726	***	0.239	Supported
H7	Deindividuation→attitude	0.156	0.066	2.37	0.018	0.147	Supported

***P < 0.001.

*B*, unstandardized coefficient; S.E., standard error; *T*, *t*-value; β, standardized coefficient.

#### Mediating effect analysis

The mediating effect of AT in the whole model was tested for significance, and the test results are shown in Table 7. If H4a is true, SNs have an indirect predictive effect on FVBI through their ATs. The indirect effect of PBC on FVBI was depicted by Bias-corrected 95% CI [0.176; 0.456] and Percentile 95% CI [0.169; 0.439], both excluding 0. Based on these statistical results, we found that AT played a mediating role between SNs and FVBI.

**TABLE 7:** Mediation effect analysis results.

Path relationship	Point estimate	S.E.	Bias-corrected 95% CI			Percentile 95% CI		
				
			Lower	Upper	*P*	Lower	Upper	*P*
Indirect effect (SN→AT→FVBI)	–0.061	0.035	–0.141	–0.002	0.040	–0.139	–0.001	0.040
Direct effect of SN on FVBI	–0.174	0.058	–0.295	–0.062	0.002	–0.290	–0.057	0.003
Total effect of SN on FVBI	–0.235	0.058	–0.353	–0.122	0.001	–0.353	–0.121	0.002
Indirect effect (PBC→AT→FVBI)	0.286	0.070	0.176	0.456	0.002	0.169	0.439	0.002
Direct effect of PBC on FVBI	0.358	0.107	0.151	0.570	0.002	0.149	0.567	0.002
Total effect of PBC on FVBI	0.645	0.089	0.468	0.810	0.003	0.470	0.812	0.002
Indirect effect (DI→AT→FVBI)	0.061	0.036	0.003	0.141	0.001	0.002	0.138	0.002
Direct effect of DI on FVBI	0.239	0.067	0.116	0.387	0.036	0.105	0.371	0.037
Total effect of DI on FVBI	0.300	0.076	0.159	0.453	0.001	0.149	0.445	0.002

Hypothesis H5a posits that PBC has an indirect effect on FVBI through AT. We described the indirect effect of PBC on FVBI by Bias-corrected 95% CI [0.176; 0.456] and Percentile 95% CI [0.169; 0.439], both excluding 0, and based on the statistical results, we also found that AT played a mediating role between PBC and FVBI.

If hypothesis H7a is true, DI has an indirect effect on the intentions toward the violent behavior of football fans through AT. Once again, we described the indirect effect of DI on FVBI by Bias-corrected 95% CI [0.003; 0.141] and Percentile 95% CI [0.002; 0.138], both excluding 0, and we found that AT played a mediating role between DI and FVBI.

## Discussion

### The direct effects of subjective norms, perceived behavioral control, attitude, and deindividuation on football fans’ violent behavioral intentions

The results showed that AT and PBC positively affect FVBI but that SNs had a negative effect. Results of the present study strongly supported previous findings which found that AT, SNs, and PBC were the antecedents of fans’ aggressive behavior (Dimmock and Grove, 2005). A stronger violent behavioral intention of football fans was correlated with a stronger violent AT, higher PBC, and lower SNs, and the AT, PBC, and SNs could effectively predict behavioral intention.

In addition, the present study also demonstrated a significant relationship between DI and FVBI. Consistent with previous similar research results (Woodhams et al., 2020), the predictive effect of DI on the intentions toward the violent behavior of football fans in China was clear. That is, the stronger the degree of DI, the stronger the intention toward violent behavior. Overall, there are at least two reasons that may explain the positive predictive effect of DI on fan violence in Chinese football. First, under the fanatical and exciting atmosphere of the stadium, fans can experience anonymous subjective cognition due to their narrow field of consciousness and sensory overload, and under the subjective cognition of anonymity, individual behaviors can become submerged by group behaviors, resulting in a decentralized sense of responsibility and less perceived constraints from laws and regulations (Postmes and Spears, 1998). Although fans act rationally at first, with the evolution and development of cluster behavior, DI can shift their decision-making toward irrationality, helping to promote the formation of violent behavioral intention. Second, the stronger the DI of fans, the more the view of individual and group invincibility will be reinforced. In a group atmosphere, highly integrated individuals influenced by the group develop a culture of hypermasculinity and their perception of self-defense and territoriality is further enhanced (Swann et al., 2012).

However, the present study suggested that AT had the greatest predictive effect on FVBI, followed by PBC, DI, and SNs. Among these, SNs were the weakest influencing factor, which was also supported by Armitage and Conner (2001). The opinions of the relatives and friends around can influence the fans’ intentions toward violent behavior to a certain extent, and indeed the stadium environment is different from the daily environment. In many ways, football spectating is a release from the pressure of daily study and work, and it is also a kind of breaking from the constraints of people, events, and responsibilities in daily life (Chen et al., 2014). This can prevent fans from paying too much attention to the opinions of their relatives and friends around them, thus weakening the predictive effect of SNs on FVBI.

### The mediating effect of attitude

The present results showed that AT played a mediating role in the relationship between SNs and FVBI. The greater the social pressure from relatives and friends, the more fans themselves will care about these opinions and ATs when they have the intention to commit violence. This social pressure is gradually internalized to conform to their expectations of themselves. In the process of constant internalization, fans’ ATs toward violent behavior can also change along with their expectations for other fans themselves, thus affecting their intentions within a group. Attitude played a mediating role in the relationship between PBC and FVBI, which supported the research of Wallace et al. (2005). PBC could predict FVBI by having an important influence on their ATs. This also suggests that the easier it is for fans to perceive the degree to which they commit violent acts, the more positive they are in their ATs toward committing violent acts and the more pronounced their violent intentions become. Therefore, one practical way to help prevent violent behavior may be to seek ways to enhance fans’ own inner perceptions of the difficulties that can stem from violent behavior.

Attitude also played a mediating role in the relationship between DI and FVBI, and in this regard our results supported the studies of Smith et al. (2007). Under the influence of DI, individual’s violent ATs can be strengthened, thus promoting the violent intentions. The reasons for this result may be 2-fold. First, DI can reduce a football fan to a simple stimulus-response organism, thereby reducing conscious self-monitoring (Prentice-Dunn and Rogers, 1980) and enhancing a fan’s positive AT toward violence. Second, the atmosphere of anonymity created by DI makes the individual AT of fans gradually shift toward the AT of the group so that violent AT of the group is exactly in line with the violent behavioral intention of the fans.

## Implications

### Theoretical implications

Compared to other countries that emphasize “individualism,” China has been influenced by the idea of “collectivism” since ancient times (Ma et al., 2016), and this idea has also influenced the culture of Chinese football fans along with the development of Chinese football. Football fan violence is closely related to the regional football fan culture, and there are differences in football fan violence in different cultures, so the discussion of football fan violence should be based on the local cultural background. Whether DI, as an evolution of “collectivism,” is a predictor of football fan violence intentions in a Chinese context that emphasizes “collectivism” is limited and requires further research to verify. Further research is needed to verify this. Our study confirmed this predictive effect, which provided guidance for reducing the occurrence of football fan violence in the context of Chinese football culture. In addition, our study extended the TPB model by combining it with DI theory for the first time in a Chinese cultural context, giving it an advantage in explaining Chinese football fans’ violent intentions. Specifically, Chinese football fans’ violent intentions are not only closely related to rational factors such as SNs and perceived behavioral control, but are also positively predicted by the irrational factor of DI in the context of Chinese “collectivism.” The extended TPB model also demonstrated the mediating role of ATs in the relationship between the factors and football fans’ violent intentions compared to the original TPB model, which was an incremental contribution to the existing knowledge.

### Management implications

In addition to theoretical contributions, this study also provided some practical insights into the management of clubs, security, and other departments. The parties involved can reduce fans’ intention to commit violence by enhancing or suppressing the four predictors in this study. For example, given the negative predictive effect of SNs on fan violence, stadium management could introduce family group attendance to enhance this social pressure from family members to exercise positive behavioral norms. Reducing the perceived behavioral control of fans is also an important aspect. By imposing a number of measures, such as banning from the stadium, fans can reinforce their self-awareness of the serious consequences of committing violent acts. For individuals who show a strong tendency to deindividualize, security departments should use video surveillance and big data screening to achieve key identification and monitoring of them. In addition, club-led community intervention will be an important part of the management strategy for football fan ATs, which can use the fan base’s enthusiasm for the club to further strengthen the propaganda in order to gradually weaken the violent ATs of the fan base.

## Conclusion and limitations

The present research showed that our comprehensive model that combines TPB and DI theory had good explanatory power for the intentions toward the violent behavior of football fans in China. Specifically, the present study revealed that SNs, PBC, DI, and AT were significantly related to FVBI. The most influential predictor wasAT, which was also significantly related to SNs, PBC, and DI as well. In addition, the present study also demonstrated that AT played a mediating role between SNs and FVBI, between PBC and FVBI, and between DI and FVBI.

This study has certain limitations that must be addressed in future studies. First, the data in the study were self-reported by football fans, so inaccuracies reported by some participants may have skewed the results to some extent. In this regard, the following measures were taken, including assuring participants of anonymity and confidentiality in completing the questionnaires, giving material reward at the end of questionnaire completion, setting a minimum length of questionnaire completion, and conducting Harman single-factor test after questionnaire collection. Although various measures have been taken to reduce their impact, the common disadvantages of self-reported questionnaires are unavoidable. Therefore, we endeavor to improve the measurement technology in follow-up research. Second, since this study used cross-sectional data, this made it difficult to infer causality in this study. Based on the good theoretical foundation provided by the hypothetical model of this study, in the future we will investigate the current model through a longitudinal design in order to provide a stronger causal interpretation of the current research model. Third, based on the TPB and DI theory, this study focused on the predictive effect of four predictors on FVBI, but the question of whether other possible external factors such as environmental factors, organizational factors, sociological factors, cultural factors, institutional factors and policy factors may directly or indirectly predict fans’ intentions to commit violence remains to be investigated. For example, the research on the relationship between the response speed of security around the stadium and the intention of violent behavior of Chinese football fans; A study on the relationship between the degree of unity of the internal organization of football fans and the intention of violent behavior of Chinese football fans. Fourth, regarding the intention of football fans to commit violence we only classified the violence in terms of the form of violence (mild and intense), while the intention of Chinese football fans to commit violence varies depending on the object on which the violence is inflicted (e.g., opposing fan groups, police, security personnel, referees, etc.). Therefore, future studies can be conducted to classify the different objects on which violence acts. Fifth, considering the relatively small sample size of this study, future studies should expand the sample size.

## Data availability statement

The raw data supporting the conclusions of this article will be made available by the authors, without undue reservation.

## Ethics statement

The studies involving human participants were reviewed and approved by the Institutional Review Board of Shandong University. The patients/participants provided their written informed consent to participate in this study.

## Author contributions

YT: data curation, formal analysis, methodology, and validation. YT, CM, and ZS: writing – original draft. All authors contributed to the article and approved the submitted version.
